# Editorial: Health and illness interactions

**DOI:** 10.3389/fsoc.2025.1561427

**Published:** 2025-02-11

**Authors:** Tracey Collett, Gayle Letherby, Louise Owusu-Kwarteng, Tanisha Spratt

**Affiliations:** ^1^School of Medicine, University of Plymouth, Plymouth, United Kingdom; ^2^School of Humanities and Social Sciences, University of Greenwich, London, United Kingdom; ^3^Centre for Death Studies, University of Bath, Bath, United Kingdom; ^4^Department of Global Health and Social Medicine, King's College London, London, United Kingdom

**Keywords:** medical sociology, sociology of health and illness, social interaction, experience of healthcare, professions of healthcare

A focus on *Health and illness interactions* is an invaluable starting point for exploring everyday, taken for granted, processes and experiences with reference to health, illness, healthcare, and public understandings of all of these (e.g., Scambler, 2018). Sociological research and theorization on such interactions has focused on lay and medical meanings/definitions and experiences and has fed directly into healthcare policy and the education of healthcare professionals. Attention has been given to, and remains on, the relationships between patients, the public and healthcare professionals (e.g., Davis, 1984; Barry et al., 2001; Scambler, 2019; McLaughlin et al., 2023), and relationships between individuals and institutions (e.g., Stacey et al., 1976/2018; Strauss et al., 1982; Abraham and Balendran, 2025). Sustained focus on health interactions has influenced public health initiatives (e.g., Goodrich and Cornwell, 2008; Silverman and Kurtz, 2017; Coulter et al., 2022) and continues to challenge the practice of healthcare. Specific concerns within the broad field of health and illness interactions include interacting with healthcare environments, diagnosis and the treatment journey, popular perceptions and misunderstandings of conditions and experiences, public health messages and political changes, and life choices and chances. Contemporary interest in health and illness interactions is generating new understandings of experiences of acute and chronic illness and disability, COVID-19 and other pandemics, antibiotic resistance, conspiracy theories, technologies, changing models of healthcare, migration and austerity.

This Research Topic contains eight articles and follows a tradition of work which acknowledges and highlights that health and illness interactions are more than just those between patients and health professionals. Three papers refer specifically to encounters between patients and experts. Scambler in *Combining experiential knowledge with scholarship in charting the decline of the national health service in England* provides a critical auto/biographical account of his own recent experiences as a patient with type 2 diabetes and subsequent polymyalgia in both primary and secondary care and deploys analytic induction to consider, and explain, his experience, against the background of the shifting nature of doctor-patient interaction occasioned by governmental politics in relation to the NHS. Scambler insists that the present impoverishment of management and care must be understood with reference to wider aspects of macro-social change. In “*Whatever I said didn't register with her”: medical fatphobia, and interactional and relational disconnect in healthcare encounters*, Kost et al. argue that medical fatphobia creates an “interactional and relational disconnect” between “fat” patients and healthcare practitioners. This leads to fatness being seen as the underlying cause of medical problems and entrenches patient and practitioner ambivalence and joint decision-making. A more positive picture is provided of patient and healthcare encounters and relationships in *Insights, beliefs, and myths surrounding tuberculosis among pulmonary patients with delayed healthcare access in a high-burden TB state in Nigeria - a qualitative inquiry*, written by Adeoye et al.. Here the authors argue that Tuberculosis (TB), as a persistent health challenge in Nigeria, necessitates more than medical intervention. TB requires a profound understanding of the diverse insights, beliefs, and myths held by patients. In this instance data from both patients and healthcare providers emphasizes a need for nuanced and culturally sensitive interventions.

The importance of relationships amongst care-givers (both formal and informal) is explored further by Stjerna and Brady and Corrigan et al.. In *Inter-embodied parental vigilance; the case of child food allergy*, Stjerna and Brady highlight the embodied aspects of parental vigilance in parenting children who have a food allergy. They argue that the lens of inter-embodiment, with a focus on bodies in relation, captures how parents' lived experience of managing food allergy intertwines with that of their children in the parent-child-health/illness triad. This embodied knowledge is often not verbalized, but Stjerna and Brady's approach offers the potential for new understandings of parent-child relations that center on chronic child health conditions. Corrigan et al.'s concern in *Integrated care systems in England: the significance of collaborative community assets in promoting and sustaining health and wellbeing* is with health policy aimed at improving health and wellbeing and reducing inequalities through local collaborative partnerships with public sector organizations, community groups, social enterprise organizations and other local agencies. The authors highlight how community groups (also known as community assets) play an important role in empowering citizens and providing much needed support to vulnerable and disadvantaged communities. However, community assets are not a substitute for functioning funded public sector services that are currently being undermined by ongoing cuts in government funding.

All authors in this special edition are concerned with the knowledge that healthcare professionals, patients, and the public hold and the impact this has on health and illness encounters. Gilbert's *Learning journeys - student learning development in the first years of a medical degree: an analysis of student conversations* explicitly focuses on undergraduate medical education and the need for the curriculum to both recognize individual learner differences and promote self-motivated, flexible, open minded, empathic lifelong learners through problem/enquiry-based curricula. The importance of experiential learning is further explored by Marks in *Invalidated and ‘Salty': an auto/biographical and theoretical review of the lived experiences of individuals with PoTS*. Drawing on her own experiences and available literature, Marks reflects on interactions between patients and healthcare providers as well as interactions between PoTSies (persons with Postural Orthostatic Tachycardia Syndrome) and those around them. Marks further examines the consequences for individuals who remain undiagnosed.

Patterson's conceptual analysis article *INTO THE WILD: Uncertain frontiers and sustainable human-nature interactions*, considers health and illness interactions between humans and the natural environment. Patterson argues that in the anthropocentric Global North political economies have historically cultivated a separation of people from nature and suggests that this separation is an eco-anomie, a damaging relational autoimmune disorder.

This special edition celebrates methodological pluralism within the field of sociology and the rich interpretations that this generates. Although a wide range of substantive topics is explored, key recurring themes include cultural perceptions of health and illness in various contexts, such as medical settings, communities, homes, and natural environments. The focus extends to both emotional and physical wellbeing, issues of inclusion and exclusion, and concepts related to the body, self, difference, and diversity in status and identity. Additionally, attention is given to the emotional management of risk. Crucially these articles demonstrate that at every level health and illness interactions do not occur in a vacuum. Rather they are bound up within personal and professional power relations although all too often this is ignored.
